# Supplementary figures and images for: Rabies transmitted from vampires to cattle: An overview
Source: PLoS One. 2025 Jan 13;20(1):e0317214. doi: 10.1371/journal.pone.0317214 (PMC11730393; doi:10.1371/journal.pone.0317214)

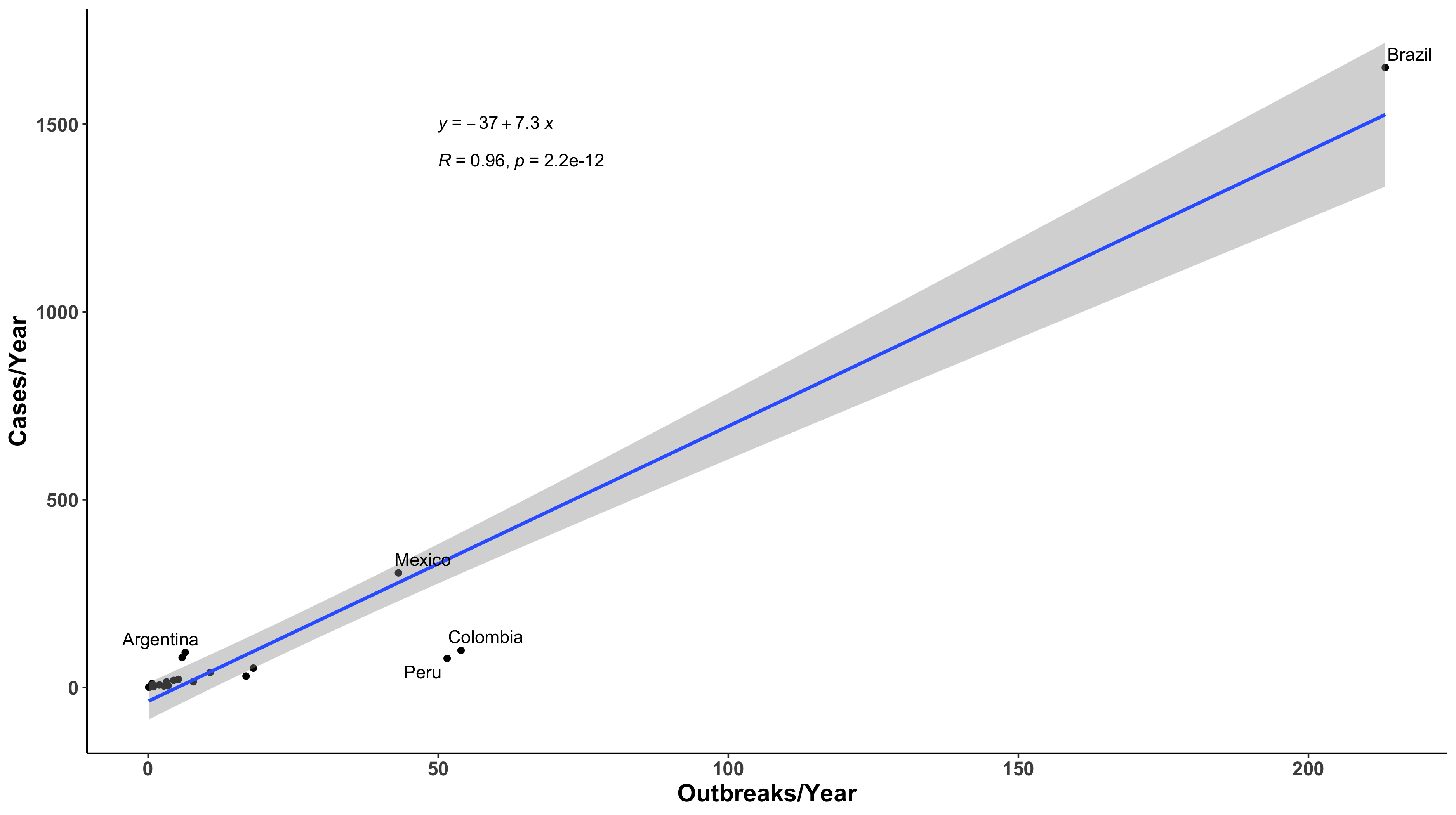

Supplement: S2 Fig — Correlation between the average annual outbreaks (x-axis) and the average annual cases of rabies in cattle (y-axis) in Latin American countries during 1970–2023. Blue line: linear model. Black spots: countries with outbreaks and cases of rabies in cattle. Statistics: R = 0.96 and p = 2.2 x 10^−12^. (TIFF) [file pone.0317214.s002.tiff]
