# Supplementary material for: Rabies transmitted from vampires to cattle: An overview
Source: PLoS One. 2025 Jan 13;20(1):e0317214. doi: 10.1371/journal.pone.0317214 (PMC11730393; doi:10.1371/journal.pone.0317214)
Supplement: S1 Table — (DOCX) [file pone.0317214.s003.docx]

# **Supporting information**

**S1 Table. Summary by country of reports of rabies in cattle and vampire bats and estimated rates.**

| **Country** | **Reports of rabies in cattle*** | **Reports of rabies in vampire bats*** | **Cattle population**** | **Human population***** | **Rate of rabies reports in cattle / 1 million cattle** | **Rate of rabies reports in vampire bats / 1 million humans** |
| --- | --- | --- | --- | --- | --- | --- |
| Argentina | 338 | 3 | 54,242,595 | 46,234,830 | 6.23 | 0.06 |
| Belize | 182 | - | 104,785 | 405,270 | 1736.89 | - |
| Bolivia | 566 | 6 | 10,739,448 | 12,224,110 | 52.71 | 0.49 |
| Brazil | 11302 | 236 | 234,352,649 | 215,313,500 | 48.22 | 1.09 |
| Chile | 9 | - | 2,955,355 | 19,603,730 | 3.04 | - |
| Colombia | 2858 | 2 | 29,642,539 | 51,874,020 | 96.41 | 0.03 |
| Costa Rica | 143 | - | 1,675,490 | 5,180,830 | 85.34 | - |
| Ecuador | 894 | - | 3,860,493 | 18,001,000 | 231.57 | - |
| El Salvador | 276 | 6 | 786,292 | 6,336,390 | 351.01 | 0.94 |
| French Guiana | 5 | - | 9,559 | 304,557 | 523.06 | - |
| Guatemala | 412 | - | 4,171,859 | 17,357,890 | 98.75 | - |
| Guyana | 35 | - | 97,832 | 808,730 | 357.75 | - |
| Honduras | 167 | - | 2,880,200 | 10,432,860 | 57.98 | - |
| Mexico | 2286 | 39 | 36,338,366 | 127,504,130 | 62.91 | 0.31 |
| Nicaragua | 102 | - | 5,612,913 | 6,948,390 | 18.17 | - |
| Panama | 233 | 1 | 1,508,571 | 4,408,580 | 154.45 | 0.22 |
| Paraguay | 961 | 5 | 13,513,375 | 6,780,740 | 71.11 | 0.73 |
| Peru | 2731 | 55 | 5,862,305 | 34,049,590 | 465.85 | 1.61 |
| Suriname | 11 | - | 34,361 | 618,040 | 320.13 | - |
| Uruguay | 48 | 1 | 11,572,000 | 3,422,790 | 4.14 | 0.29 |
| Venezuela | 310 | 6 | 16,644,150 | 28,301,700 | 18.62 | 0.21 |

*Source SIRVERA: <https://sirvera.panaftosa.org.br/>

**Source cattle population: <https://www.fao.org/faostat/es/#data>

***Source human population: <https://data.worldbank.org/indicator/SP.POP.TOTL>
