# Supplementary material for: Rabies transmitted from vampires to cattle: An overview
Source: PLoS One. 2025 Jan 13;20(1):e0317214. doi: 10.1371/journal.pone.0317214 (PMC11730393; doi:10.1371/journal.pone.0317214)
Supplement: Attachment — Submitted filename: Considerations PONE-D-24-15035.pdf [file pone.0317214.s004.pdf]

Considerations

I consider that this manuscript provides useful information and quantified data that can be considered to improve the epidemiological surveillance of rabies in Latin America.

Some of my comments and suggestions are:

In the introduction, line 118, the authors should rewrite the objective and review whether the appropriate word evaluate could be modified to identify or determine the association between the number of D. rotundus infected with RABV and confirmed cases of rabies in the livestock in Latin America.

They also mention that they are considering the sustained expansion of agriculture in this region and nowhere in the manuscript do they mention how they considered it, I did not find at least figures, or territorial extensions that show cases in areas with this factor. If this was considered in the study, it is necessary to add it to Materials and methods.

line 158, you need to add a bibliographic reference in the text that supports the described methodology and add it to the references section

In line 414, the authors justify the association between rabies outbreaks in D. rotundus and cattle with the density of cattle, however they do not refer to a figure for the number of animals that could strengthen the idea. Furthermore, the authors could consider that this association is most likely due to the small number of samples from D. rotundus sent to the laboratory. In this case, it is important to refer to whether the campaign for the control and surveillance of rabies in livestock species has as its strategy the control of populations in D. rorundus, or whether it would be an area to strengthen in Peru and Colombia.

lines 417 and 418. Review the citations in the text and approve them according to the journal's guidelines.

In the Materials and Methods section, specify whether only cases in cattle or also in other livestock species were considered and put cattle in parentheses so that the study population is better understood.

In the materials and methods part, define what was considered a case and what outbreak. Because otherwise the information will be misinterpreted and considered a duplicate, which makes it difficult to understand the graphs in Suplementary Figure 1.

In accordance with the objective set by the authors, it is recommended to add a descriptive table similar to table 1 but with data on the cases that occurred in D.rorundus or those detected with RABV infection, as well as adding a footer to the table in both cases that defines the source of the information and the Diagnostic test they used to confirm them as positive for the disease.

It is necessary to add the conclusions section, it seems that lines 454 to 463 refer to them, however, here it is also necessary to restructure the paragraph to explain how this analysis can be useful to determine where and when should prioritize the monitoring of D. rotundus due to having populations infected with RABV and the probable epidemiological trends of rabies in the near future in Latin America.

In the results section, although the authors mention Figure 1, they do not describe the interpretation of the distribution. Here a paragraph is necessary that states what is observed or makes a comparison between them. It is highly recommended to add a map with the name of the countries to better understand which geographical areas it refers
