# Supplementary material for: Rabies transmitted from vampires to cattle: An overview
Source: PLoS One. 2025 Jan 13;20(1):e0317214. doi: 10.1371/journal.pone.0317214 (PMC11730393; doi:10.1371/journal.pone.0317214)
Supplement: Attachment — Submitted filename: Response to Reviewers.docx [file pone.0317214.s005.docx]

**Response to Reviewers**

**PONE-D-24-15035**

Below is shown each comment from the Academic Editor and the reviewers (black), and after each one the response from the authors (blue).

**Academic Editor Comments**

1. Please ensure that your manuscript meets PLOS ONE's style requirements, including those for file naming. The PLOS ONE style templates can be found at

https://journals.plos.org/plosone/s/file?id=wjVg/PLOSOne_formatting_sample_main_body.pdf and

<https://journals.plos.org/plosone/s/file?id=ba62/PLOSOne_formatting_sample_title_authors_affiliations.pdf>.

**Authors' response:** The suggested links have been re-checked and the manuscript meets the style requirements of PLOS ONE.

2. Thank you for stating in your Funding Statement: “LEE was supported by National Science Foundation CAREER (2235295) and HEGS (2116748) awards. Research reported in this publication was supported by the National Institute of Allergy and Infectious Diseases of the National Institutes of Health under Award Number K01AI168452. The content is solely the responsibility of the authors and does not necessarily represent the official views of the National Institutes of Health.” Please provide an amended statement that declares *all* the funding or sources of support (whether external or internal to your organization) received during this study, as detailed online in our guide for authors at http://journals.plos.org/plosone/s/submit-now.  Please also include the statement “There was no additional external funding received for this study.” in your updated Funding Statement. Please include your amended Funding Statement within your cover letter. We will change the online submission form on your behalf.

**Authors' response:** The manuscript and data submitted on the website were reviewed, and all funds or sources of support received during the study were included; additionally, “No additional external funding was received for this study” was included in the statement. The modification of the financing statement was included in the cover letter, as follows: LEE was supported by National Science Foundation CAREER (2235295) and HEGS (2116748) awards. Research reported in this publication was supported by the National Institute of Allergy and Infectious Diseases of the National Institutes of Health under Award Number K01AI168452. The content is solely the responsibility of the authors and does not necessarily represent the official views of the National Institutes of Health. No additional external funding was received for this study.

3. We note that Figure 1 in your submission contain [map/satellite] images which may be copyrighted. All PLOS content is published under the Creative Commons Attribution License (CC BY 4.0), which means that the manuscript, images, and Supporting Information files will be freely available online, and any third party is permitted to access, download, copy, distribute, and use these materials in any way, even commercially, with proper attribution. For these reasons, we cannot publish previously copyrighted maps or satellite images created using proprietary data, such as Google software (Google Maps, Street View, and Earth). For more information, see our copyright guidelines: <http://journals.plos.org/plosone/s/licenses-and-copyright>. We require you to either (1) present written permission from the copyright holder to publish these figures specifically under the CC BY 4.0 license, or (2) remove the figures from your submission:

a. You may seek permission from the original copyright holder of Figure 1 to publish the content specifically under the CC BY 4.0 license.

We recommend that you contact the original copyright holder with the Content Permission Form (http://journals.plos.org/plosone/s/file?id=7c09/content-permission-form.pdf) and the following text:

“I request permission for the open-access journal PLOS ONE to publish XXX under the Creative Commons Attribution License (CCAL) CC BY 4.0 (http://creativecommons.org/licenses/by/4.0/). Please be aware that this license allows unrestricted use and distribution, even commercially, by third parties. Please reply and provide explicit written permission to publish XXX under a CC BY license and complete the attached form.”

Please upload the completed Content Permission Form or other proof of granted permissions as an "Other" file with your submission.

In the figure caption of the copyrighted figure, please include the following text: “Reprinted from [ref] under a CC BY license, with permission from [name of publisher], original copyright [original copyright year].”

b. If you are unable to obtain permission from the original copyright holder to publish these figures under the CC BY 4.0 license or if the copyright holder’s requirements are incompatible with the CC BY 4.0 license, please either i) remove the figure or ii) supply a replacement figure that complies with the CC BY 4.0 license. Please check copyright information on all replacement figures and update the figure caption with source information. If applicable, please specify in the figure caption text when a figure is similar but not identical to the original image and is therefore for illustrative purposes only.

The following resources for replacing copyrighted map figures may be helpful:

USGS National Map Viewer (public domain): http://viewer.nationalmap.gov/viewer/

The Gateway to Astronaut Photography of Earth (public domain): http://eol.jsc.nasa.gov/sseop/clickmap/

Maps at the CIA (public domain): https://www.cia.gov/library/publications/the-world-factbook/index.html and https://www.cia.gov/library/publications/cia-maps-publications/index.html

NASA Earth Observatory (public domain): http://earthobservatory.nasa.gov/

Landsat: http://landsat.visibleearth.nasa.gov/

USGS EROS (Earth Resources Observatory and Science (EROS) Center) (public domain): http://eros.usgs.gov/#

Natural Earth (public domain): http://www.naturalearthdata.com/

**Authors' response:** The map in Figure 1 is not previously copyrighted and is not a satellite image created with data from Google software (Google Maps, Street View, and Earth), it is a map created by the authors in R and RStudio, using shapefiles from the sources suggested by PLOS ONE journal.

**Reviewers' Comments**

1. Is the manuscript technically sound, and do the data support the conclusions?

Reviewer #1: No

Reviewer #2: Yes

**Authors' response:** We reviewed and adjusted the manuscript according to the comments of the Academic Editor and the reviewers and considered that the manuscript describes technically sound scientific research with data supporting the conclusions, the analyses were done rigorously, and the conclusions were generated from the results presented.

2. Has the statistical analysis been performed appropriately and rigorously?

Reviewer #1: Yes

Reviewer #2: I Don't Know

**Authors' response:** We confirm that the statistical analysis was carried out appropriately and rigorously

3. Have the authors made all data underlying the findings in their manuscript fully available?

Reviewer #1: Yes

Reviewer #2: No

**Authors' response:** The data used in the analysis for this manuscript are completely available without restrictions, on the SIRVERA website: https://sirvera.panaftosa.org.br/. In addition, the R code used for the analyses will be deposited in a public repository (like GitHub: https://github.com/) and will also be delivered by email without restrictions by the authors to anyone who requests them.

4. Is the manuscript presented in an intelligible fashion and written in standard English?

Reviewer #1: No

Reviewer #2: Yes

**Authors' response:** Comments on English from the reviewers were considered, the English of the manuscript was reviewed by members of the Virginia Tech laboratory (United States) and the Grammarly tool was used to improve the English.

5. Review Comments to the Author

**Reviewer #1:**

1. In the introduction, line 118, the authors should rewrite the objective and review whether the appropriate word evaluate could be modified to identify or determine the association between the number of *D. rotundus* infected with RABV and confirmed cases of rabies in the livestock in Latin America.

**Authors' response:** The objective of the manuscript was modified according to the reviewer's comment.

2. They also mention that they are considering the sustained expansion of agriculture in this region and nowhere in the manuscript do they mention how they considered it, I did not find at least figures, or territorial extensions that show cases in areas with this factor. If this was considered in the study, it is necessary to add it to Materials and methods.

**Authors' response:** The sustained expansion of agriculture in the region was not considered either methodologically or analytically, but this text is included followed by the objective to justify it and is supported with a reference.

3. Line 158, you need to add a bibliographic reference in the text that supports the described methodology and add it to the references section.

**Authors' response:** References 30 and 31 were included.

4. In line 414, the authors justify the association between rabies outbreaks in *D. rotundus* and cattle with the density of cattle, however they do not refer to a figure for the number of animals that could strengthen the idea. Furthermore, the authors could consider that this association is most likely due to the small number of samples from *D. rotundus* sent to the laboratory. In this case, it is important to refer to whether the campaign for the control and surveillance of rabies in livestock species has as its strategy the control of populations in *D. rorundus*, or whether it would be an area to strengthen in Peru and Colombia.

**Authors' response:** S1 Table with the quantitative data that supports Figure 4 was included as Supporting information. In addition, the analysis was run again to adjust the statistical values and include El Salvador in the text. In addition, text was included in the Discussion, as suggested, supported by reference 21.

5. Lines 417 and 418. Review the citations in the text and approve them according to the journal's guidelines.

**Authors' response:** The citations were reviewed and comply with the journal's guidelines.

6. In the Materials and Methods section, specify whether only cases in cattle or also in other livestock species were considered and put cattle in parentheses so that the study population is better understood.

**Authors' response:** In lines 136 and 141 it is indicated that the data are for rabies in cattle.

7. In the materials and methods part, define what was considered a case and what outbreak. Because otherwise the information will be misinterpreted and considered a duplicate, which makes it difficult to understand the graphs in Suplementary Figure 1.

**Authors' response:** In lines 148 and 149 it is indicated that it is a case and that it is an outbreak.

8. In accordance with the objective set by the authors, it is recommended to add a descriptive table similar to table 1 but with data on the cases that occurred in *D. rotundus* or those detected with RABV infection, as well as adding a footer to the table in both cases that defines the source of the information and the Diagnostic test they used to confirm them as positive for the disease.

**Authors' response:** Some of the additional data suggested by the reviewer was included in Table S1 of the Supporting information. The source of the data in Table 1 is included at the end of that table's label, and the source of the data in the S1 Table is included at the end of the table with asterisks.

9. It is necessary to add the conclusions section, it seems that lines 454 to 463 refer to them, however, here it is also necessary to restructure the paragraph to explain how this analysis can be useful to determine where and when should prioritize the monitoring of D. rotundus due to having populations infected with RABV and the probable epidemiological trends of rabies in the near future in Latin America.

**Authors' response:** The Conclusions subtitle was included, and that section was enriched according to the reviewer's comment.

10. In the results section, although the authors mention Figure 1, they do not describe the interpretation of the distribution. Here a paragraph is necessary that states what is observed or makes a comparison between them. It is highly recommended to add a map with the name of the countries to better understand which geographical areas it refers.

**Authors' response:** The most relevant part of Figure 1 is included in lines: 165-173, 184-188, and 405-406. The names of the countries were included in the maps in Figure 1.

**Reviewer #2:**

1. Line 50 - Why do the authors use the term "spillover"? It might be better to consider transmission throughout the entire manuscript.

**Authors' response:** The word "spillover" was changed to "cross-species transmission" throughout the manuscript.

2. Lines 52-54 – The value of the association should be included, as well as the incidence.

**Authors' response:** The suggested values were included in the indicated lines.

3. Line 60 – Are there epidemiological analyses to support the mentioned risk factors? The values of the analyses should be included. If not, it is speculative and should be either removed or only discussed.

**Authors' response:** Some of these factors are discussed in the Discussion.

4. Line 81 – It's not clear what the impacts on the environment would be. The authors need to clarify.

**Authors' response:** This comment was answered on line 88, with their reference.

5. Lines 85-86 – Transmission methods need to be clarified in addition to the virus elimination route.

**Authors' response:** Lines 91 and 92 were included with their reference.

6. Line 96 – There is also an important role of wild animals as virus reservoirs.

**Authors' response:** Text was included on lines 102 and 103, along with references.

7. Line 104 – In Brazil, there has also been a recent increase in the detection of bat variants in humans.

**Authors' response:** In addition, the detection of Text and references were included in lines 113 and 114 to respond to this comment. has recently increased.

8. Line 109 – Standardize the use of "D. rotundus" (abbreviated) after the first mention in the introduction.

**Authors' response:** The use of “*D. rotundus*” (abbreviated) after the first mention in the introduction was revised and standardized, only leaving the full name if it starts a paragraph or if named after a point in a paragraph.

9. In the introduction, I suggest the authors include data on the economic impacts of rabies resulting from its occurrence in production animals (cattle).

**Authors' response:** A paragraph about the economic impact was included between lines 120 and 129.

10. Line 140 – I would like to understand how notifications are entered into SIRVERA. Who provides this regional data? Who receives the notifications and forwards them to the Pan American Health Organization? The logistics of obtaining and including data need to be clear to readers.

**Authors' response:** In lines 151 to 153 what was commented by the reviewer was expanded.

11. I would also like to understand in the methodology how the authors calculated the incidence values mentioned in the abstract. Was there a collection of the population size of the herds in the evaluated areas? Or were only the case numbers considered without taking into account the size of the cattle herds in the areas to consider true incidence?

**Authors' response:** This comment was clarified in lines 173 to 177.

12. In Figure 1A, I see countries inconsistent with the scale presented. Only two colorations? What about the others? Why only include the extremes (larger and smaller)? I would like to visualize the data in its entirety through the map. Also, the image definition is quite poor for visualization.

**Authors' response:** New maps were made for Figure 1, improving the color palette, and the resolution or quality of the figure was improved.

13. Lines 190-194 – It should not be considered as seasonal variation since it only considers the time of year (month) without taking into account the other characteristics that determine seasonality.

**Authors' response:** Changed the word "seasonal" to "monthly" on line 220.

14. Line 228 – In which species?

**Authors' response:** The species was included in line 254.

15. Line 297 – It would be important to discuss the mandatory vaccination of cattle in different countries.

**Authors' response:** The text and references were included in lines 331 to 336.

16. Line 351 – Reference 30 also shows the importance of sanitary education actions, as well as population control of hematophagous bats and vaccination as tools for rabies control in herbivores.

**Authors' response:** The suggested reference was included on line 393.

17. Lines 372-374 – There are works in Brazil that have evaluated the cost-benefit effect of applying vaccines for rabies control in herbivores (<https://doi.org/10.1590/1678-5150-PVB-6201>).

**Authors' response:** The text and the suggested reference on lines 416 and 417 were included.

18. In the discussion, I would also like to see information on population control measures of hematophagous bats, such as the application of vampiricidal pastes. It is known that these actions have good efficacy but can also pose a high risk to non-hematophagous bat species. A discussion on these actions is important to include.

**Authors' response:** A paragraph on this topic was included between lines 496 and 507.

19. Lines 405; 417-418 and others – Adjust the reference to the number.

**Authors' response:** Adjustments were made to the numbering of references.

20. Lines 423-424 - The existence of rabies monitoring programs in bats should be discussed. Which countries do this? Is there a requirement for this monitoring? The topic can be better detailed.

**Authors' response:** Text and references were included to respond to this comment on lines 474 to 483.
