# Supplementary material for: Rabies transmitted from vampires to cattle: An overview
Source: PLoS One. 2025 Jan 13;20(1):e0317214. doi: 10.1371/journal.pone.0317214 (PMC11730393; doi:10.1371/journal.pone.0317214)
Supplement: Attachment — Submitted filename: Response to Reviewers_V2.docx [file pone.0317214.s006.docx]

**Response to Reviewers**

**PONE-D-24-15035R1**

**Reviewer #3 Comments**

Many points are not discussed as: 1) subnotification of cases, 2) accidental cases of rabies in humans transmitted by *D. rotundus* and 3) data from each studied country.

**Authors' response:**

Many thanks to reviewer #3 for his valuable comments, a review of the entire manuscript was made, and adjustments were made throughout the text (see attached manuscript).

The three main comments are answered below:

1) We agree with the reviewer regarding the subnotification of cases. We apologize for missing this key factor regarding the patterns presented in the article. We have incorporated new text addressing the effect of the subnotification of cases in the results and overall conclusions. We also updated the discussion and present a new rationale about the likely effects of the underreporting of rabies cases in Latin America. Finally, we present potential alternatives to and future research to address the subnotification in future studies.

The new text is included below (lines 527 to 570 of the manuscript):

“Underreporting of cattle rabies cases in Latin America can be a major concern, influenced by factors such as surveillance limitations, lack of awareness, and socioeconomic conditions. Existing rabies surveillance systems often rely on passive reporting by livestock farmers, which can lead to underreporting in remote areas [63]. Local livestock farmers and veterinarians may lack training to identify rabies, resulting in missed cases in aeras where the disease is not endemic [64]. In rural Latin America, economic pressures may discourage reporting by farmers to avoid potential economic losses [39]. Limited access to veterinary services in rural areas may hamper timely diagnosis and reporting [47]. Low awareness of rabies in some areas contributes to underreporting [65]. We argue that education and training should be conducted in the areas identified in this study. Improved education and training of livestock farmers and veterinary staff are key to improving reporting rates [65].

Additionally, underreporting of bovine rabies cases in Latin America has significant implications for public health, economic stability, and disease management strategies. Underreporting leads to an underestimation of livestock deaths, with studies suggesting that for every reported case, there may be 4.6 unreported cases [16]. Economic losses attributed to bovine rabies can reach up to $171,992 per year in specific regions, significantly affecting smallholder farmers [16]. The persistence of rabies in livestock, particularly from *D. rotundus*, poses a direct risk to human health, as evidenced by the high percentage of human cases linked to rabies in livestock [65]. Lack of consistent reporting hampers the ability to track rabies outbreaks, complicating public health responses [66]. Underreporting of cases leads to poorly informed vaccination strategies, which may not adequately address the true scale of the problem [46,67]. Improved surveillance and reporting mechanisms are essential to accurately assess the disease burden and implement effective control measures [65].

Future research to address underreporting of bovine rabies cases in Latin America should focus on improving surveillance systems, enhancing farmer education, and using advanced modeling techniques. Transitioning from passive to active surveillance methods to ensure timely reporting of cases, as seen in Ecuador's AGROCALIDAD program [63]. Similarly, implementing GIS tools to map and analyze spatiotemporal trends of rabies transmission [47]. Educating farmers about rabies symptoms and reporting protocols could help increase case identification [16]. Involving local communities in surveillance efforts to improve reporting rates, particularly in remote areas, can help detect cryptic cases of wildlife rabies [16].”

2) We agree with the reviewer in our intentional overlook of accidental cases of rabies in humans transmitted by *D. rotundus*. Accidental cases of rabies in humans transmitted by *D. rotundus* were not discussed because our intention with this manuscript was to present the status quo on passive epidemiology about bovine rabies. We intentionally left human cases out because this is under study as part of another PhD thesis chapter at the Escobar lab. We, however, agree with the reviewer in that this is a very interesting topic that deserves attention. To address this suggestion, we have cited literature regarding accidental human cases and have expanded our discussion accordingly.

The new text is included below (lines 711 to 718 of the manuscript):

“*Desmodus rotundus* can pose a serious threat to public health, particularly in rural and indigenous communities where interactions with bats are more frequent. For example, in 2011, an outbreak in Yupicusa, Peru, resulted in the deaths of 21 children and two adults, highlighting the severe impact of *D. rotundus*-transmitted rabies on vulnerable populations [8]. A recent outbreak among the Maxakali people in Brazil resulted in the death of four children due to recreational contact with bats, highlighting cultural practices that increase the risk of exposure [87].”

3) We apologize for our limited elaboration on the data from each studied country in the previous version of the manuscript. We have revised the paper following the reviewer’s observation. A new discussion text was included to discuss in more detail incidence and prevalence values at the country level. In addition, rabies reports in cattle and vampire bats and estimated rates are summarized by country in Table S1 of the supporting information.

The new text is included below (lines 587 to 618 of the manuscript):

“The prevalence of rabies in cattle in Brazil, Colombia, Mexico, and Peru can be attributed to interrelated factors, such as the distribution of *D. rotundus*, cattle density, and environmental conditions. These countries present ecological and socioeconomic characteristics that facilitate sustained rabies transmission among cattle. The density of *D. rotundus* populations correlates with rabies outbreaks [69]. Brazil and Colombia have significant populations of *D. rotundus*, which thrive in rural areas where cattle are abundant [47]. High cattle populations in these countries increase the risk of rabies transmission. For example, Colombia reported 4888 confirmed cases of cattle rabies between 2005 and 2019, with significant outbreaks in regions with high cattle density [47,52]. Rabies cases in Mexico were concentrated in states with high cattle densities, indicating a direct relationship between cattle density and outbreak frequency [69]. Climatic and topographic conditions in these countries create favorable environments for *D. rotundus*, enhancing its interaction with cattle [69]. Socioeconomic factors influence inadequate vaccination coverage and limited public awareness, which contribute to the persistence of rabies outbreaks despite control efforts [52,67].”
